# 

**Published:** 1902-10-11

**Authors:** 


					the hospital. "The standard of highest purity."?Lancet. Oct. hth, 1902.
0 CADBURY'S m* U ?. CADBURY'S
COCOA rl Jpi m COCOA
ABSOLUTELY PURE. rTl _J HO DRUGS OR ALKALIES U8BB.
^0. 837. Vol. XXXIII. KwS?peBr.]
Saturday, Oct. 11th, 1902.
[anbse^tioDjCTm.,] PRICB TWOPENCE.

				

